# Cadmium toxicity and treatment: An update 

**DOI:** 10.22088/cjim.8.3.135

**Published:** 2017

**Authors:** Mehrdad Rafati Rahimzadeh, Mehravar Rafati Rahimzadeh, Sohrab Kazemi, Ali-akbar Moghadamnia

**Affiliations:** 1 Department of Nursing, Babol University of Medical Sciences, Babol, Iran.; 2 Department of Medical Physics, Kashan University of Medical Sciences, Kashan, Iran.; 3 Cellular and Molecular Biology Research Center, Health Research Institute, Babol, Iran.; 4 Neuroscience Research Center, Health Research Institute, Babol University of Medical Sciences, Babol, Iran.

**Keywords:** Cadmium, Poisoning, Decontamination, Nanoparticles, Chelating agents

## Abstract

Cadmium poisoning has been reported from many parts of the world. It is one of the global health problems that affect many organs and in some cases it can cause deaths annually. Long-term exposure to cadmium through air, water, soil, and food leads to cancer and organ system toxicity such as skeletal, urinary, reproductive, cardiovascular, central and peripheral nervous, and respiratory systems. Cadmium levels can be measured in the blood, urine, hair, nail and saliva samples. Patients with cadmium toxicity need gastrointestinal tract irrigation, supportive care, and chemical decontamination traditional-based chelation therapy with appropriate new chelating agents and nanoparticle-based antidotes. Furthermore it has been likewise recommended to determine the level of food contamination and suspicious areas, consider public education and awareness programs for the exposed people to prevent cadmium poisoning.

Over a few decades ago, to follow the abundant accessibility of various chemical materials, the rate of intoxication has amazingly increased (1, 2). People may use some drugs and chemicals in the wrong way, as a result they may be poisoned intentionally or accidentally (3, 4). Heavy metals similar to other poisonous chemicals, from natural or industrial sources, can pose serious threats to human life (5). Cadmium (Cd, atomic number 48, atomic mass number 112, melting point 321 °C, and boiling point 765°C) is an element with soft, ductile, silvery white with bluish color, lustrous, and electropositive properties. It does not have any odor or taste, and is very poisonous. Cd has eight stable isotopes:^ 106 ^Cd,^ 108^ Cd, ^110^ Cd, ^111 ^Cd, ^112^ Cd, ^113^ Cd, ^114^ Cd, and ^116 ^Cd. The most common isotopes are ^112^ Cd and ^114^ Cd (6). Cadmium also forms a variety of complex organic amines, sulfur complex, chloro complexes, and chelates. Cd ions form soluble salts of carbonates, arsenates, phosphates, and ferrocyanide compounds. Accompanying zinc production, it can be produced in different commercial forms. It is used as alloys in electroplating (auto industries) and in production of pigments (cadmium sulfate, cadmium selenide), likewise as stabilizers for polyvinyl plastic, and in batteries (rechargeable Ni-Cd batteries) (6, 7). 

**Epidemiology: **In spite of the dramatic worldwide production, consumption and release of Cd compounds in the environment show no efficient recycling way for them. Accordingly, human exposure to Cd compounds may create a serious health problem. Cadmium has been used in nickel-cadmium battery, as a pigment in paint production, likewise, in electroplating and producing polyvinyl chloride plastic. Furthermore, cadmium is present in most foodstuffs, and depending on dietary habits, its level varies greatly. 

Cadmium considerably exists in environment, as a result of human activities, such as the use of fossil fuels, metal ore combustion and waste burning. Leaking sewage sludge to agricultural soil may cause the transfer of cadmium compounds adsorbed by plants that may play a significant role in food chain, and accumulate in various human organs. Also, the other great source of cadmium exposure is cigarette smoke. When cadmium was measured in smokers’ blood samples, it showed that they had 4-5 times cd levels in blood higher than the non-smokers (8). 

Exposure to cadmium in many different ways has been reported during the past century. Damage to the lungs in Cd-exposed workers was reported as early as the 1930s. Moreover, in the next decades, some bone and kidney toxicity cases of cadmium exposure were described. After World War II, in the 1960’s and 1970’s, Japanese people suffered from different levels of pollution. Itai-itai disease was one of these conditions caused by chronic cadmium contaminated rice fields. The number of patients affected by the disease was estimated around 400 patients from 1910 to 2007 (9). 

Another international collaborative study in 16 European countries has reported that the amount of cadmium in mother- child couples exceeded the tolerable weekly intake. In that study, Poland had the highest urine-Cd in comparison between the16 countries while Denmark showed the lowest level (10). In the United States, approximately 600 tons of Cd compound are produced every year and 150 tons are imported from other countries (11). 

While most parts of Iran, rice and wheat are the daily staple food. Iranian farmers in achieving high quality crops may have applied enormous amount of phosphate fertilizers and sludge waste, which consequently contains higher concentration of cadmium. This may increase Cd absorption via consumption of foods produced in crops. 

Based on FAO/WHO rules the permitted level of cadmium in rice is 0.2 mg/kg (12). The result showed Iranian rice samples had higher level of Cd than the permitted concentration. In addition, the risk will increase consuming other sources such as farm products (vegetables) and sea foods (fish, etc), if cadmium contamination occurs (13).

Nowadays, cadmium exposure has decreased in many countries (14), but it has a very long biological half-life (10-30 years) (10) and human activities related to cadmium should be restricted to a minimal or no harmful level (10).

It is necessary to prepare the basic information of cadmium poisoning and design an educational and prophylactic plan to substantially reduce the incidence of its toxicity. The present review may be informative and helpful to achieve the purpose of managing all aspects of cadmium compound poisoning.

**Mechanism of Toxicity:** Cadmium affects cell proliferation, differentiation, and apoptosis. These activities interact with DNA repair mechanism, the generation of reaction oxygen species (ROS) and the induction of apoptosis (15). Cadmium binds to the mitochondria and can inhibit both cellular respiration and oxidative phosphorylation at low concentration (16). 

It results in chromosomal aberrations, sister chromatid exchange, DNA strand breaks, and DNA- protein crosslinks in cell lines. Cadmium causes mutations and chromosomal deletions potentially (17). Its toxicity involves depletion of reduced glutathione (GSH), binds sulfhydryl groups with protein, and causes to enhance production of reactive oxygen species (ROS) such as superoxide ion, hydrogen peroxide, and hydroxyl radicals. Cadmium also inhibits the activity of antioxidant enzymes, such as catalase, manganese-superoxide dismutase, and copper/zinc-dismutase (18). Metallothionein is a zinc – concentrating protein that contains 33% cysteine. Metallothionein also can act as a free- radical scavenger. It scavenges hydroxyl and superoxide radicals (19). Generally, the cells that contain metallothioneins are resistant to cadmium toxicity. On the other hand, the cells that cannot synthesize metallothioneins are sensitive to cadmium intoxication (20). Cadmium can modulate the cellular level of Ca^2+ ^and the activities of caspases and nitrogen-activated protein kinases (MRPKs) in the cells, in which these processes cause apoptosis indirectly (21). 

While P_53 _causes cell death by directly binding to mitochondrial membrane proteins. Expression of B-cell lymphoma-extra-large (Bcl-xl), which is a transmembrane molecule in the mitochondria, suppresses mitochondrial-mediated apoptosis and enhances cancer cells. To address the challenge to the observation posed; binding of P_53_ to Bcl-xl can inhibit protein and apoptotic cell death (22). 

Cadmium can induce ROS production and result in oxidative stress. This mechanism may express the role of cadmium in organ toxicity, carcinogenicity and apoptotic cell death (fig1).

**Fig 1:**
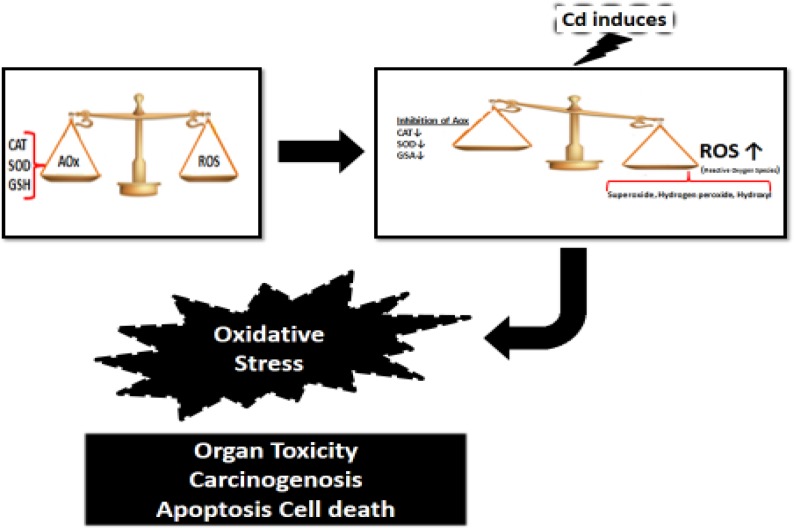
Effect of Cd induces and reactive oxygen species (ROS) in human body

**Clinical Manifestation:** Different forms of cadmium compounds have different clinical manifestations and toxic effects that were explained in the details below.

**Cadmium bone and Itai-itai disease:** Several studies mentioned cadmium can affect the skeletal system. Exposure to cadmium caused skeletal demineralization, whereby it may directly interact with bone cells, diminish mineralization, also inhibit procollagen C-proteinases and collagen production (22). Clinical findings associated with osteoporosis include pain, physical impairment, and decreased quality of life. Besides, decreased bone density imparts increased risk for bone fractures. Osteoporotic fractures are most common in post-menopausal women that can result to disability. Pseudofractures following osteomalacia and severe skeletal decalcification may be observed as well (23). 

When serum PTH levels decreased with higher cadmium exposure, this may induce the release of calcium from bone tissue (24). Cadmium may interact with metabolism of calcium, vitamin D_3_ and collagen. Therefore osteomalacia or osteoporosis could be observed in delayed manifestations of severe cadmium poisoning (22).

Itai-itai disease is the most severe form of chronic cadmium intoxication. The first recognition occurred in Jinzu river, Toyama Prefecture, Japan (25). Two hypotheses have been proposed to explain bone lesion. Direct actions of cadmium on bone include; disappearance of metaphyseal trabeculas and shortened epiphyseal cartilage in which cadmium caused osteoporotic, but does not observe osteomalacic changes via radiographical controls. Indirect effects of cadmium on bone include; thinning bone cortex, trabecular bone loss, in addition there is a decrease in number of osteocytes and acid mucopolysaccharides in epiphyseal cartilage (25). Cadmium intoxicants cause femoral and low back pain in initial manifestation, the further pain spread to the other areas of the body. Moreover, skeletal deformities can cause bone fractures (26).

**Renal damage in cadmium toxicity:** Cadmium predominantly accumulates in kidney and liver, but it can be found in other tissues such as bone and placenta. It has been reported that occupational and environmental exposures to cadmium have implicated renal dysfunction (27). Cadmium exposure can show early signs of renal damage, proteinuria, calcium loss and tubular lesion. Urine analysis may help to prove early signs of renal damage (16). Generally, the glomerular filtration rate (GFR) and reserve filtration capacity will be diminished, and severe cadmium toxicity may induce nephrotoxicity with complications such as; glucosuria, aminoaciduria, hyperphosphaturia, hypercalciuria, polyuria and decreased buffering capacity (28). Cellular damage and functional integrity in proximal tubules resulted in loss of calcium, amino acids, enzymes, and increase proteins in the urine. On the other hand, a decreased tubular reabsorption of a few molecular-weight proteins, lead to tubular proteinuria. The most common proteins in urine are beta 2-microglobulin, retinol- binding protein and alpha 1-microglobulin (29).

**Cadmium and reproductive system:** Several previous studies found that cadmium has the potential to affect reproduction and development in several mammalian species, and recent studies have also confirmed these findings (30). Compared to animal studies, it is claimed that cadmium decreases density, volume and number of sperms, and increases immature sperm forms (31). These problems are followed by a defect in spermatogenesis, sperm quality, and secretory functions of accessory glands. Besides, it decreases libido, fertility, and serum testosterone level (32). In female reproductive system, the function of ovary and development of oocytes may be inhibited. Steroidogenesis is reduced under Cd toxicity and ovarian hemorrhage and necrosis can co-occur (30). It has been reported that the rate of spontaneous abortion and time of pregnancy are increased and the rate of live births decreased (31).

**Cadmium and cardiovascular system:** In vitro studies have indicated the involvement of cadmium in endothelial dysfunction as well as carotid intima-media thickness (IMT). Moreover, the formation of atherosclerotic plaques were promoted in vivo (33). Following cadmium intoxication, endothelial dysfunction at starting of cardiovascular disease (CVD), loss of endothelial cell structure causing cell death, and thrombogenic events may occur. These results support the hypothesis cadmium involvement in cardiovascular disease and myocardial infarction (34). Epidemiologic studies had shown the association of cadmium exposure with risk of high blood pressure (systolic and diastolic blood pressures). 

Cadmium may inhibit endothelial nitric oxide synthase and suppresses acetylcholine induced vascular relaxation resulting in hypertension (35). It may stimulate production of cytokines and induce endothelial damage. These mechanisms cause atherogenesis and long- term exposure may increase the incidence of peripheral arterial disease (36). Cadmium toxic exposure may increase cardiovascular mortality (37).

**Cadmium and other systems:** The acute central and peripheral neurotoxicity of cadmium has been recently reported (38). Cadmium may also induce cellular damage and lipid peroxidation in brain. Its effect on monoaminoxidase (MAO) is responsible for oxidative deamination of monoamine neurotransmitters (38). Cadmium increases production of free radicals in CNS and decreases cellular defense against oxidation (39) . In general, the outcomes of this mechanism are olfactory dysfunction, neurobehavioral defects in attention, disorder in psychomotor activity, and memory (40). Poisoning may lead to neurodegenerative disorders, such as Parkinson, Alzheimer, and Huntington’s diseases accompanying with loss of memory and behavioral changes.

Recent study has shown a possible involvement of cadmium in pulmonary diseases such as chronic obstructive disease and emphysema (41). Animal studies showed that cadmium chloride can decrease lung vital capacity and increase alveolar wall thickness. Inhalation of cadmium as vapor in the absence of antioxidants, and condition of oxidative stress, may result in pulmonary inflammation and emphysema (41). According to the Agency for Toxic Substances and Disease Registry (ATSDR) suggestion; cadmium is a possible lung carcinogen in humans (41). 

Cadmium is absorbed through the gastrointestinal tract (GIT). Its solubility and absorption are affected by gastric and/ or intestinal pH. In fact, cadmium reacts with HCl and forms of cadmium chloride. It can induce the inflammation of GIT. The H_2_ blockers can raise gastric pH, causing to decrease the solubility and inhibit the absorption of cadmium (42). Several studies had shown cadmium can induce liver damage in acute stage. Prolonged oral cadmium ingestion can cause Itai-itai disease in chronic phase (43).

Limited research studies in cadmium poisoning with skin manifestations showed hyperkeratosis and acanthosis, accompanied with occasional ulcerative change, and an increase of the mitotic index of the skin cells (44). 

**Cadmium and carcinogenicity:** Cadmium compounds were categorized as carcinogenic in humans by International Agency for Research on Cancer (IARC) (45). It may be considered as lung carcinogen, also inducer of prostatic or renal cancers .The important point is that cadmium can disorder testosterone production and induce testicular interstitial cells hyperplasia (46). Some reports suggested that cadmium may be involved malignancies of liver, hemotopoitic system, bladder and stomach (47). Furthermore, cadmium may be a potential risk factor for breast cancer. Another study suggested that cadmium exposure may be involved in pancreas cancer because of inducing increased risk for neoplasia (47).

The cellular and molecular mechanisms implicating cadmium carcinogenicity include the activation of proto-oncogenes, inactivation of tumor suppressor genes, disruption of cell adhesion, and inhibition of DNA repair (48). In fact, DNA strand damage or DNA-protein crosslinks disorder may completely cause to inhibit cell growth. In summary, it is suggested that cadmium exposure can affect cell proliferation, differentiation, apoptosis, cell signaling and other cellular activities. These activities could bear on carcinogenesis directly or indirectly (47).

**Diagnostic evaluation:** Cadmium levels in blood, urine, hair and nails samples are often determined in paraclinic lab tests.

**Urine:** Kidneys are the main organ to be affected by cadmium in long term exposure (49). Crinnion suggested; urinary cadmium concentration equal or greater than 0.5 µg/g creatinine is associated with renal damage, also the concentrations more than 2.0 µg/g of creatinine may be translated into extensive damage (50). 

Tubular dysfunction followed by cadmium nephrotoxicity increases urinary excretion of low molecular weight proteins such as ß_2_-microglobulin, α_1_ microglobulin, retinol binding protein, enzymes such as N - acetyl - ß – glucosaminidase, and calcium (51). In this situation, sensitive tests (low molecular weight proteinuria) may be positive and mixed proteinuria (low and high molecular weight proteins excretion in urine) is seen (28). 

**Blood:** Long cadmium half-life (30 years) may be due to long term accumulation of cadmium in the body but the short half-life of cadmium in blood (three to four months) could have result in a recent exposure. The limit of detection for blood cadmium concentration is 0.3 µg/L (52). Blood Cadmium was measured by two techniques; either electrothermal atomic- absorption spectrophotometry or the inductively coupled plasma mass spectrometry. Based on the research studies done in the National Health and Nutrition Examination Surveys (NHANES), the values at or below the limit of detection of cadmium in all of participants are follows: 1999-200: 0.3µg/l; 2003-2004: 0.14µg/l; 2005-2010: 0.2µg/l; (53). 

**Hair–nail and saliva:** Determination of the trace element levels in hair and nails is the subject of interest in biomedical sciences (54). Trace elements accumulate in the body in a long time may affect biomedical and metabolic processes over time (55). Additionally, the sampling, transport and storage of hair and nails samples are easy and feasible and analysis of trace elements in the samples is cheap and fast (55).

Cadmium accumulates in body for a long time and its concentration can gradually increase several years after exposure .The levels of cadmium in the hair have different reference values of various countries e.g., in Italy is 0.03 mg/kg, England 0.11 mg/kg, and in Japan 0.05 mg/kg(55). Further, it is reported that the amount of cadmium in hair is 0.61±1.13 µg g^-1^ and the nails 1.11±0.83 µg g^-1^ elsewhere (56). Saliva analysis can be an excellent method for long term detection of heavy metal contamination. The mean level of cadmium in saliva with tolerable standard limit in human body is less than 0.55 µg/l (57).

**Application of nanomaterial in the diagnosis of cadmium poisoning:** Nanomaterials have different applications such as tissue and organ engineering, medical instruments, drug delivery, diagnosis evaluation, prevention and management (58). Utilizing nanotechnology for diagnosing and eliminating toxic metals such as cadmium can help to manage cadmium intoxication and increase environment safety (59). 

Several nanoparticles have been used for diagnostics. One of the nanoparticles is quantum dots (QDs). QDs are made of fluorescent labels of cadmium selenide or zinc sulfide. When cadmium poisoning occurs, it is released and entered into cells containing zinc ions. Capping QDs with ZnO effectively prevents cadmium formation, and achieving better to cover material is done. A gene expression test helped to determine this coating (60).

**Treatment of cadmium poisoning**

**Immediate considerations:** After evaluation of the airways, breathing and circulation, protection and care is necessary. The GIT should be irrigated to remove cadmium containing solutions. Acute or chronic ingesting of cadmium salts is rare, but it may lead to death. The lowest lethal dose of Cd is 5 gr in a 70 kg man. If emesis has not occurred, gastric lavage is performed soon. A small nasogastric tube tube must be used (61). Activated charcoal cannot effectively absorb the metal.

Hospitalization may help the patients exposed to cadmium for evaluating the extent of liver damage, gastrointestinal, urinary and respiratory tracts thus, we suggest supportive therapy (61). 

**Natural and chemical decontamination:** Industrial and mining activities may release cadmium ions in waste water. Natural decontamination can be introduced using some medicinal plants. The seeds of *Moringa oleifera*, peanuts (*Arachis hypogaea*), cowpeas (*Vigna unguiculata*), urad (*Vigna mungo*) and corn (*Zea mays*) were used for water purification. These seeds can absorb and neutralize colloidal positive charges. This action causes to absorb the negative charged impurities and metals in waste water (62). 

Some plants are used for phytomediation to extract and detoxify some pollutants. They have ability to accumulate heavy metals such as; Cd, Cr, Pb, Co, Ag, Se and Hg in their tissues. For example, *Cleome Gynandra* has been used as a phytoorigin detoxifier (63). Phytochelating activity has an important role in metal detoxification by the sequestration of Zn and Cd (64).

The removal of heavy metals from contaminated soil includes; 1) washing, leaching, flushing with chemical agents, 2) adding some non- toxic materials to reduce solubility of heavy metal 3) electromigration, 4) covering the original pollutants with clean materials, 5) mixing polluted materials with clean materials in surface and subsurface to reduce the concentration of heavy metals, and 6) phytoremediation by plants (65). The absorption yield depends on different factors such as; pH of environment, ionic power, and metal concentration in solution or biomass. These factors can affect biological storage, biogeochemical migration and toxic properties of heavy metals (66).

**Chelating agents**

**Ethylenediaminetetraacetic acid (EDTA):** EDTA significantly increased urinary elimination of cadmium. One important point is that EDTA may increase Cd content in the kidneys and may increase the risk of renal dysfunction (67). Normal dose of EDTA is 500 mg of Ca^2+^ EDTA in combination with 50 mg/kg of glutathione (GSH) via IV infusion over the next 24 hours and repeated over 12 consecutive days (68). Renal dysfunction could be reversed if its initial urine cadmium concentration is <10 µg/gr of creatinine. Urine cadmium concentration more than 10 µg/gr of creatinine may induce irreversible renal damage (67).

**Penicillamine (DPA):** Penicillamine used to reduce toxic concentrations of mercury and lead exposure, is not efficient in cadmium overdose (69).

**Dimercaprol:** Dimercaprol [British anti- Lewisite (BAL)] is efficient antidote in heavy metal poisoning (70). BAL and their analogues meso-2, 3-dimercaptosuccinic acid DMSA and 2, 3-dimercapto-1-propanesulfonic acid DMPS are used as antidote course of therapy for heavy metal poisoning. 

BAL must be administered in the first 4 hours of poisoning. Deep intramuscular injection of a dose 3-4 mg/kg in gluteal muscle is recommended. It is given every 4 hours for the first two days, and twice daily for the next 10 days (71). It has been reported that cadmium-BAL complex has more nephrotoxic effects than cadmium alone (28) and previously mentioned that the combination is not helpful (72) and it is recommended to treat or manage actual poison exposure with other treatments. Possibly, BAL therapy may increase the risk of nephrotoxicity (73). In addition, BAL increases kidney and liver cadmium burdens, may decrease survival and enhances nephrotoxicity. For these reasons, it is not given in cadmium intoxication. 

**Dithiocarbamates:** Dithiocarbamate derivatives (fig 2) have been used in many fields such as; agriculture, manufacturing, and medicine (74). N- tetramethylene dithiocarbamate (ATC) is one of derivatives of dithiocarbamates with chelating action. It enhances the urinary and biliary excretion of cadmium, also reduces the side effects and general symptoms of poisoning. It may be useful for primary diagnostic evaluation of the efficacy of chelating agents (75). The efficacy of dithiocarbamates has been confirmed in reducing cadmium toxicity in animal studies (61). There is a necessity for the administration of these chelating agents in humans to be documented.

**Fig 2:**
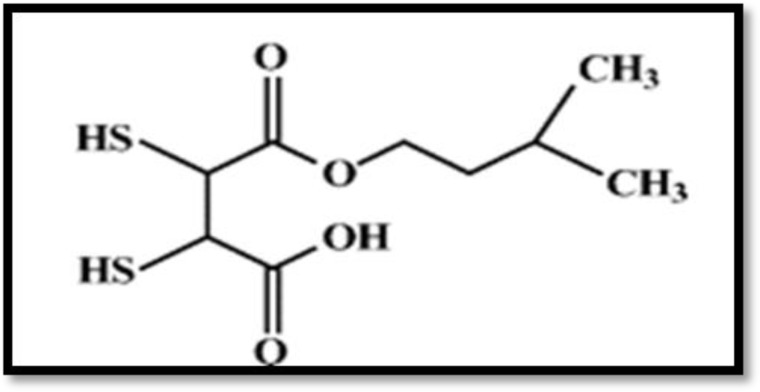
Dithiocarbamate ammonium pyrrolidine dithiocarbamate or tetramethylene dithiocarbamate

**Meso 2, 3-dimercaptosuccinic acid (Succimer, DMSA):** It is a water-soluble analogue of BAL, with chemical formula C_4_H_6_O_4_S_2_ (76). Tolerable dose of DMSA is10 mg/kg, three times a day (61) but it is not an intracellular chelator. Cadmium binds tightly to metallothionein and stores in liver and kidneys. In consequence, it seems that DMSA cannot be a drug of choice in cadmium poisoning (16). 

**2, 3- dimercapto-1-propane sulfonic acid (Unithiol,** **DMPS):** It is a water soluble analogue of BAL with chemical formula C_3_H_7_O_3_S_3_Na. It is available in different dosage forms as oral, intravenous, rectal, or topical (76). DMPS is transported into intracellular space. It has not shown major adverse effects (77). DMPS is oxidized to disulfide form. At least 80% of DMPS is oxidized within the first 30 min and 84% of total DMPS is excreted by the kidneys within 96 hours (78). Dose: 5 mg/kg intravenously 4 hourly for 24 hours, and may be increased to 100 mg twice a day, if needed.

**New DMSA analogues:** DMSA mono and diesters are more effective and safe antidotes for heavy metal poisoning compared to DMSA alone (79). Among these monoesters, *monoisoamyl *DMSA* (MiADMSA)*, a C_5_ branched alkyl monoester (fig 3) was shown to be effective for lead, cadmium, mercury and gallium arsenide overdose (80). MiADMSA is a water- soluble, lipophilic chelating agent. It can enter intracellularly and access to different endogenous ligands. Consequently MiADMSA is more preferred than its parent compound (80). 

**Fig 3:**
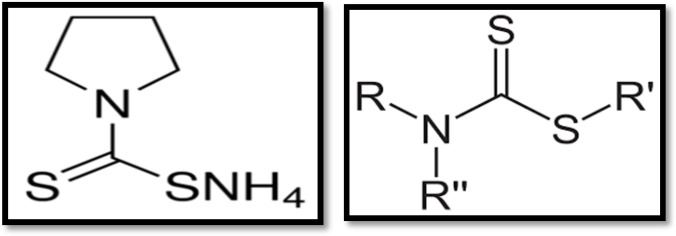
Strutural formula of MiADMSA (mono isoamyl ester of dimercaptosuccinic acid)

MiADMSA can enter into cell and bind to intracellular cadmium. Because of the effects of antioxidants, cadmium-induced oxidative stress is delayed due to the presence of MiADMSA (79).

*Monomethyl* DMSA *(MmDMSA)* and *Monocyclohexyl *DMSA *(MchDMSA) are the other DMSA analogues (*fig 4*). They *are lipophilic compounds and can penetrate into cells. They are efficient after oral administration and may reduce the whole body cadmium levels following its overdose (79). 

**Fig 4:**
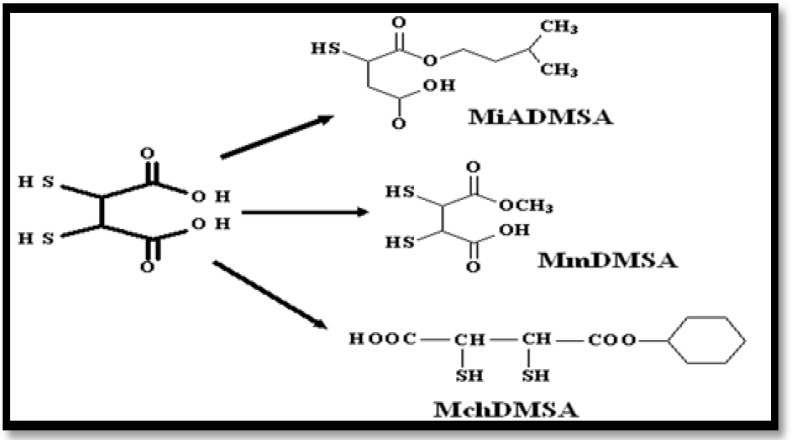
New monoesters of dimercaptosuccinic acid (DMSA)

**Combination therapy with chelating agents and other substances:** Combination therapy is an effective route in the management of heavy metal toxicity (3). Optimal effects of chelating agent therapy may be achieved when combination of DMSA and MiADMSA is administered (77). A combination of DMSA and calcium trisodium diethylene triaminepentaacetate (CaDTPA) has been effectively used in acute oral cadmium. These two agents reduce cadmium concentration and toxic effect in the body (81). It has been found that N-acetyl cysteine (NAC) and DMPS reduced cadmium – induced hepatic and renal metallothionein Also, NAC may increase the efficacy of DMPS (82).

Some reports have shown that antioxidants like vitamin C and vitamin E have protective effect against cadmium induced toxicity in different experimental animals (83). Combination of ascorbic acid, alpha-tocopherol, and selenium can be effective against cadmium toxicity in rat. As a result, lipid peroxidation increased and glutathione levels decreased in the intestine of rats. This combination showed a protective effect of the combination against cadmium toxicity in intestine (84). Indeed, vitamins A, C, E, and selenium can prevent or reduce many toxic effects of cadmium on some organs and tissues such as liver, kidney, skeleton, and blood. The other elements are zinc and magnesium with many clinical applications. It has been suggested that zinc facilitates immune function and prevent free radicals. Magnesium is an essential cofactor to activate many enzyme systems in humans. Zn and Mg can reverse Cd- induced renal toxicity. Cadmium toxicity causes to decrease antioxidant enzymes, produces reactive oxygen species, and lipid peroxidation. In fact, Zn and Mg can confront reactive oxygen species and lipid peroxidation(85). Chelating agents for cadmium poisoning are ongoing, and may produce a new agent that is accessible, safe and effective, without aggravating end-organ. Overall, there is no evidence to justify the use of any chelator regarding treatment of cadmium toxicity.

**Application of nanoparticle in the treatment of cadmium poisoning:** Cadmium can be adsorbed by Al_2_O_3_ nanoparticles. Generally, Al_2_O_3_ nanoparticles are appropriate for removing Zn and Cd from solution/sorbent systems. Al_2_O_3_ nanoparticles with low citrate concentrations are used to remove Cd and Zn from contaminated solutions (86). Carbon nanotubes (CNTs) remove metal ions from aqueous solutions (87). Cadmium can be removed from wastewater by nanosized TiO_2_ particles (88). 

**Plasma exchange-hemodialysis-plasmapheresis:** Plasma exchange may have started 24-36 hours after the appearance of clinical signs and symptoms, when life-threatening toxicity happened and the health team could not choose any alternative treatment. Plasma exchange must only be used in emergency situations. Hence, it can potentially be helpful in heavy-metal toxicity (89).

 Hemoperfusion and hemodialysis are not useful in the treatment of cadmium poisonings. Furthermore, cadmium is eliminated very differently, it has very low residual renal function and inefficient cadmium removal via dialysis. In severe renal damage, hemodialysis has benefits in replacing kidney function (90). Some of the toxic substances can strongly bind to plasma proteins and cannot be removed through hemodialysis. Plasmapheresis is practical and sensible to remove protein- bound heavy metals in plasma. Nonetheless, there are no controlled studies on plasmapheresis in any specific intoxication (91). 

In Conclusion, Cadmium compound poisoning leads to harmful effects on various organs and systems. It is considered as a potential worldwide threat to environment and human being. It transports via air, water, soil, and food chain, etc. There are risks for human health from exposure to cadmium compounds. Cadmium intoxications need decontamination via GIT irrigation, supportive care, and chemical decontamination, the use of nanoparticles, traditional and new chelating agents and combination therapy.

It is recommended to identify the individual’s highly sensitive people to cadmium exposure, and ensure any contamination of agricultural soils, drinking water and food chain. It is necessary to pay attention to the handling of cadmium compounds and it is then suggested to detect the contaminated sites and design education and awareness programs for the potential at risk population to minimize cadmium toxicity. 
